# Intense or Spatially Heterogeneous Predation Can Select against Prey Dispersal

**DOI:** 10.1371/journal.pone.0028924

**Published:** 2012-01-11

**Authors:** Frederic Barraquand, David J. Murrell

**Affiliations:** 1 Centre d'Etudes Biologiques de Chizé, CNRS, Villiers-en-bois, France; 2 Université Pierre and Marie Curie - Paris 6, Paris, France; 3 Dep. of Arctic and Marine Biology, University of Tromsø, Tromsø, Norway; 4 Department of Genetics, Environment and Evolution, University College London, London, United Kingdom; 5 CoMPLEX, University College London, London, United Kingdom; National Research Council of Italy (CNR), Italy

## Abstract

Dispersal theory generally predicts kin competition, inbreeding, and temporal variation in habitat quality should select for dispersal, whereas spatial variation in habitat quality should select against dispersal. The effect of predation on the evolution of dispersal is currently not well-known: because predation can be variable in both space and time, it is not clear whether or when predation will promote dispersal within prey. Moreover, the evolution of prey dispersal affects strongly the encounter rate of predator and prey individuals, which greatly determines the ecological dynamics, and in turn changes the selection pressures for prey dispersal, in an eco-evolutionary feedback loop. When taken all together the effect of predation on prey dispersal is rather difficult to predict. We analyze a spatially explicit, individual-based predator-prey model and its mathematical approximation to investigate the evolution of prey dispersal. Competition and predation depend on local, rather than landscape-scale densities, and the spatial pattern of predation corresponds well to that of predators using restricted home ranges (e.g. central-place foragers). Analyses show the balance between the level of competition and predation pressure an individual is expected to experience determines whether prey should disperse or stay close to their parents and siblings, and more predation selects for less prey dispersal. Predators with smaller home ranges also select for less prey dispersal; more prey dispersal is favoured if predators have large home ranges, are very mobile, and/or are evenly distributed across the landscape.

## Introduction

Dispersal is the glue which holds local populations together, enabling the re-colonisation of patches after local extinction, and maintaining gene flow between populations. However, whilst dispersal may be important to the viability of populations, it is essentially driven by natural selection on individuals. Understanding the often conflicting selection pressures behind individual dispersal behaviour is therefore an important question in ecology and evolution. A considerable body of dispersal theory is devoted to that aim [1]–[3], and it highlights two main evolutionary forces behind the selection for dispersal. On the one hand are factors pertaining to the genetic structure of the population, namely kin competition and inbreeding avoidance [4]; and on the other hand, the spatio-temporal variability of habitat quality, both biotic and abiotic.

Temporal variation in habitat quality usually selects for increased/longer dispersal because a good location now is likely to decline in quality. Temporal variation in habitat quality can be driven by abiotic factors (e.g. climate), but many biotic demographic factors contribute to temporal variability, such as chaotic population dynamics [5], or demographic stochasticity [6], and these can also select for dispersal [7]. In contrast, spatial variability in habitat quality is normally thought to select against dispersal because individuals strive to stay in high quality patches [8]–[10]. What dispersal strategies are selected in dynamic landscapes with both spatial and temporal variation in individual fitness is less clear, because selective pressures for and against dispersal are intermingled; and the degree of correlation in the temporal or spatial variation influences these main results. For example, Travis [11] found that increased spatial autocorrelation of environmental variation tends to select for greater dispersal, and increased temporal correlation for a decrease in dispersal. The evolution of dispersal in spatiotemporally variable environments has therefore been, and still is, the focus of much of the theoretical research (e.g. [9], [12] and [13]). North et al. [13] showed that increased spatial correlation of habitat quality (leading to larger patches in their model) can indeed select for increased dispersal, but only as as long as the patches are not initially quite small. When habitat quality is fine-grained, increased spatial autocorrelation in habitat quality may actually select for a decrease in dispersal distance [13].

Here, we investigate a somewhat less studied but logical driver of dispersal: predation. Weisser [14] suggested that predation could have contrasting effects on dispersal depending on the dispersal phase affected. For example, predation might select for dispersal in order for prey individuals to leave an area with high predation pressure; or it might select against dispersal if predators increase the cost of dispersal by eating individuals that are in transit. However, it is not clear that predation can select for dispersal when there is spatial variation in predation risk (which is the rule rather than the exception in natural systems). First, individuals located in low predation risk habitats could be better off not dispersing at all because, unless dispersal is completely conditional on predation risk and prey movements are perfectly tuned to avoiding predators, moving prey also take the risk of landing closer to a predator. Second, individuals located in high predation risk habitats, while benefiting from their escape, might be too few to direct evolution towards higher dispersal rates as they are very likely to be eaten before reproducing. These two arguments point out there might be selection for low prey dispersal when predation is strong and if prey do not have much information about the spatial variation of predation risk (the case we consider here, i.e. unconditional dispersal). Lack of information about predators may happen in prey species that do not possess sophisticated cognitive abilities (e.g. most invertebrates), but may happen as well in cognitive prey if predators manage prey vigilance [15]. Moreover, the spacing patterns of predators, their home range size, and the resulting spatial pattern of predation pressure are very likely to influence the selection pressures. For instance, if predators are very clumped it might make sense not to move and stay in refuges (even ephemeral ones), but if predation pressure is uniformly or evenly distributed in space, it may be advantageous for a prey to disperse to avoid local competition with kin.

Here, we attempt to clarify the effect of predation on the evolution of dispersal strategies. Our model is inspired by the ecology of carnivorous birds and mammals (e.g. raptors, carnivorans), as these predators often prey on herbivores (e.g. rodents, ungulates) within a restricted home range whose size is smaller than the spatial extent of the predator population. Predator home ranges are in those cases only partially overlapping, thereby generating a spatial heterogeneity in predation rates, especially if the activity is more intense close to a central place such as as nest or den. In the most simple case, when predators have a fixed home range, and their reproduction is either slow or maintained by alternative prey (very generalist predators), predator population size and spatial pattern can be considered constant in time. Predation is then equivalent to a density independent, spatially varying mortality risk. The outcome of selective pressures is a balance between the relative intensities and spatial scales of competition processes that select for dispersal; and predation processes that, as we shall show below, generally select against dispersal, especially when the predation spatial pattern is spatially heterogeneous (e.g. because predator home range sizes are small).

The specific effects of predation (rather than just density-independent or environmental mortality) become clearer when we allow for different spatial patterns in the predator population (aggregated, uniform, segregated); different predator movements that generate a more or less autocorrelated spatiotemporal pattern of mortality; and finally population dynamics feedbacks between the prey and predator population.

Dispersal, as it has been often remarked, can be separated into natal and breeding dispersal [16]. Here, we focus on the evolution of dispersal rate (adult dispersal), for a fixed dispersal kernel, although similar results can be obtained for the natal dispersal range and are presented in Figure S2. We show that more predation generally selects against dispersal; and a more spatially heterogeneous predation pressure, generated by smaller predator home ranges or more clustered predator nest patterns, also selects against dispersal. Finally, we show that natural selection on the prey does not necessarily lead to the common good (large prey population sizes) when predators have a numerical response, i.e. we observe conflict between the individual-level and population-level which leads to a tragedy of the commons scenario [17].

## Methods

In the following, we study the evolution of prey dispersal in response to predation, starting from a spatially explicit individual-based model (IBM) and deriving population-level equations [18], [19] on which we perform invasion analyses [20]. The population-level model is akin to Lotka-Volterra equations with localized predation, localized dispersal, and localized competition.

### Individual-based model

We start with an IBM that tracks individual prey and predators that are located as points (as opposed to patches on a lattice), with continuous x-coordinates, on a homogeneous landscape having periodic (or wrap around) boundaries. The boundary conditions effectively mean the landscape is large compared to the scales of competition, and predation, and edge effects are not important to the ecological or evolutionary dynamics. For most of the results, we assume the landscape to be one-dimensional, but we also show that the results are qualitatively matched in a two dimensional landscape. A continuous timeline is assumed, so individuals overlap in their generations; and at any point in time an individual may undergo a birth, death, predation, or movement event (described below and in Appendix S1) which may be dependent on the local abundance of other prey and predators. These assumptions mean that any spatial variation in prey and predators is due purely to the interactions between, and movement of the individuals in the community. The resulting model is called a dynamic spatial point process [18], [19].

**Figure 1:**
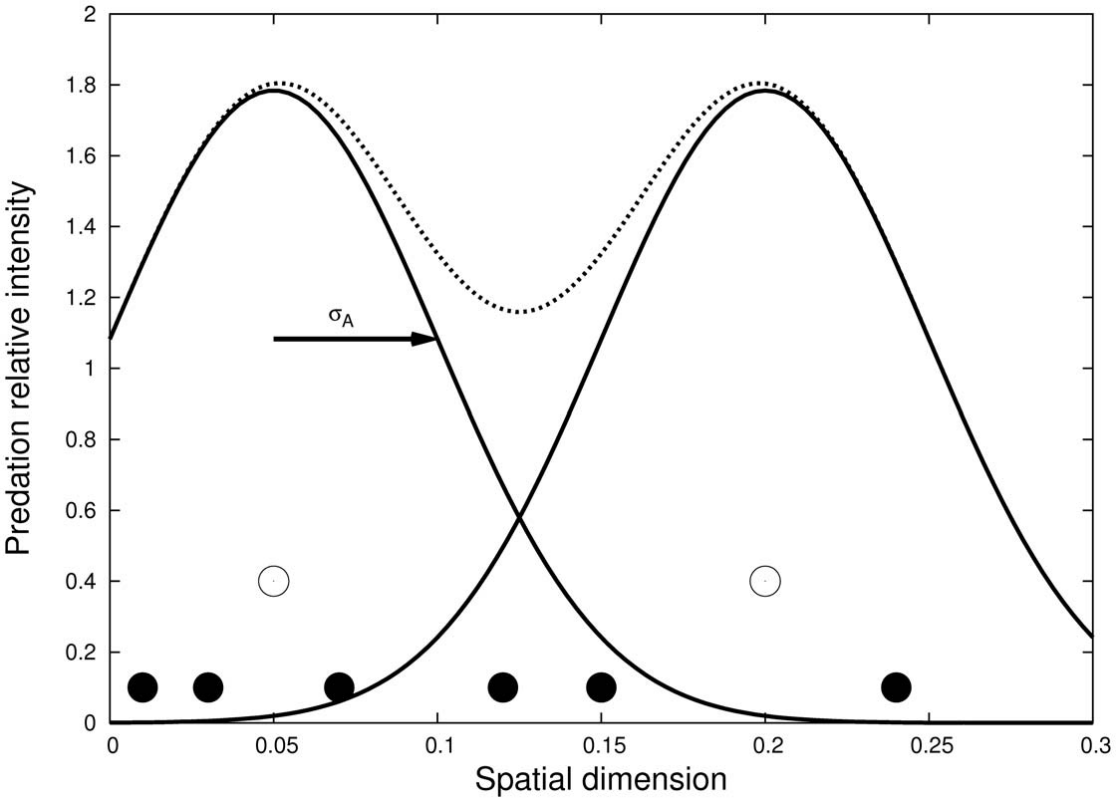
Schematic depiction of predation in the individual-based model. Predators are pictured as open circles while prey individuals are represented as black dots. Thick black lines represent the spatial distribution of foraging effort for each predator (i.e. the probability density of attack as described by kernel *a* in the model), while the dotted line, which is the sum of the black curves, represent the relative predation risk for the prey. 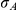, the spatial dispersion of the foraging effort distribution, is referred to in the main text as the predator home range size. On average, predators tend to kill more prey in the center of their home range so that prey progressively concentrate at predator home range boundaries; and this creates a negative spatial correlation between predator and prey distributions.

Prey individuals have a fixed fecundity, and produce single prey newborns at a rate 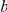; which means interbirth durations are exponentially distributed with mean 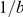. Density independent mortality occurs at a rate 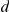, which implies again that the expected lifetime of an isolated prey individual is 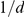; and that in the absence of predation and competition its expected lifetime reproduction output is 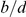. Prey density dependent mortality occurs from adding up contributions from each neighbouring prey individual. Each neighbour contributes to the death of an individual by the per capita competition term, 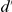, which is weighted by the competition kernel 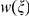 describing how the per capita competition strength is affected by the distance 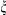 between individuals in the pair. The competition kernel 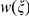 is a probability density function, and in the results below we use a Gaussian function with mean 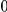, and scale parameter 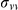. This means competition is most intense between individuals that are nearby in space, and the scale parameter determines how quickly the kernel declines with distance. Small values for 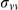 indicate competition is very intense between nearby neighbours, but quickly drops off to zero; whereas large values for 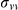 indicate competition is less intense between nearby neighbours, but attenuates more slowly with increasing distance (the mathematical details of the model are presented in [21] and Appendix S1). The predation part of the model is modeled in a similar way; more specifically, the model assumes that prey per capita mortality generated by any predator, 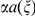, decreases with increasing distance 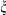 to the predator centre of activity according to the kernel 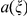, and this is summed over all predators. This means death from predation is most likely to be caused by the nearest predator to a prey individual, but there is also a small probability that the death is caused by a more distant predator (see Figure 1). The spatial scale of the attack kernel 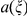, that can be thought of as predator home range size, is denoted 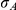. Small home ranges (small 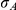) therefore concentrate the predator's foraging effort around a central location such as a nest, meaning attack rates are high near to the nest but drop off quickly with increasing distance; whereas large home ranges (large values for 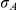) have a more even foraging effort that covers a larger area. The impact of predation upon prey individuals is depicted in Figure 1 and Figure S3.

At the point of birth prey move with an average dispersal distance 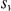 (also denoted dispersal range) from their parent, according to a Gaussian kernel. Subsequently, an adult prey has a constant dispersal/movement rate 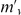, which amounts to the assumption that adult dispersal events occur every 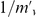 timesteps on average, and at each movement event the adult moves the same average distance as at birth (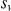). Distances moved are sampled according to a Gaussian kernel, which means movements are neither directional nor in any way density dependent. We perform invasion analyses on this IBM [22], on both movement traits (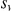 and 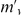), which requires the introduction of a resident dispersal rate 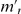, as well as a mutant dispersal rate 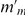 (in Figure S1 and Figure S2, additional invasion analyses using dispersal distances 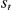 and 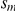 are performed). In results using two-dimensional landscapes, all kernels are bivariate Gaussian, and retain the same single scale parameters.

In the simplest case, predators are assumed to be present in constant numbers (no predator births or deaths), which corresponds well to systems where predator demography happens on a time scale that is much longer than that of the prey. It might also represent very generalist predators, sustaining themselves with other abundant resources when the prey considered in the model is scarce, and whose birth and death rates are therefore almost unaffected by focal prey abundance, even though they contribute to prey depletion. This assumption is later relaxed when we consider predator birth rate to be linked to prey attack (i.e. a numerical response is implemented), allowing for population dynamics feedbacks between the predator and prey compartements of the model. In this second model, each prey attack by a predator can lead to a predator birth with probability 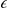, so that 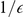 prey items are needed on average to produce a new predator. Newborn predators are displaced at a distance 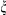 from their parents according to a Gaussian kernel 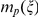 with spatial scale 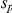. Furthermore, in both models (constant or variable predator numbers), adult predators can move, using the same movement kernel, at a rate 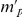. Table 1 lists the model parameters.

**Table 1:** Model parameters with their default values.

Parameter type	Parameter name	Symbol	Reference value	Unit
Per capita	Attack efficiency	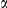	0.002	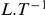
rates	Prey productivity	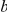	0.4	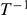
	Prey density independent death rate	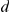	0.1	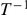
	Prey competition strength	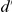	0.0005	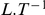
	Predator density independent death rate	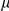	0.01	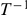
	Prey competition strength	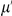	0	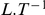
	Prey dispersal rate	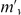	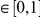	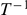
	Predator movement rate	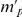	0	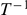
Interaction and	Prey dispersal range	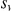	0.05	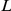
movement	Prey competition range	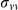	0.05	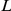
distances	Predator attack range ( = Home range size)	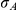	0.05	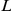
	Predator movement range	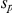	0.05	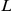
	Predator competition range	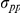	0.05	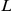
Other	Predator conversion efficiency	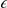	0.05	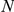

Default values are used in the paper unless mentioned otherwise in the figure captions. Units: L = [Length], T = [Time], N = [No unit]. Note that rate and distances parameters are not independent in their effects on spatial structure: rates determine large-scale densities, and these densities govern the meaning of distance parameters, i.e. with how many competitors and predators can a prey individual interact.

Note that a predator point, which is best interpreted as the home range center of a predator individual, can also be thought of as a small social group, i.e. the predator point might be the nest location of a pair of birds, or the den of Carnivore pack, around which predation is distributed according to the kernel 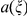 [23].

In the absence of predator demography, the environment from the prey point of view is a spatial pattern of predation pressure, or a ‘predation risk landscape’, and our current model then resembles closely that of Bolker [12] or North et al. [13]. Depending on the predator home range size, which here is equivalent to the average predator foraging distance, the predation pattern will be more or less uniform, as shown in Figure 1. Such variation in the spatial predation pattern can also be due to changes in the location of predators home range centers (e.g. nests), for which we envisage three kinds of spatial distributions: clumped, uniform (random), and segregated. Increasing both the segregation of predator nests, and the size of their home range will lead to a more even spatial pattern of predation pressure (hunting effort) across the landscape.

### Moment equations

It is relatively straightforward to implement the above processes into a computer algorithm (see Appendix S1), and certainly much can be understood by running realisations of the IBM with different parameter sets [24]. However, it is also possible to mathematically derive the expected trends in population dynamics and spatial structure, and the associated selective pressures for dispersal; this allows for more detailed analyses.

Using a master equation approach [19], and taking expectations of probabilistic event rates, one can compute the dynamics of expected landscape densities (first moments) as well as the dynamics of the expected spatial structure in the population (as summarised by the so-called second moments). The second moment 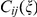 describes the expected density of pairs of individuals of species 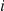 and 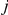 separated by a distance 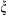. For now we make the assumption that the predator population is of constant size (see section above). The equation for the expected prey population dynamics is then
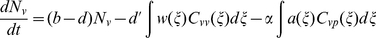
(1)


The first term in eq. 1 accounts for the change in density due to density independent births and deaths. The second term takes into account additional deaths caused by competition between neighbouring prey. It is computed as the expected density of prey pairs 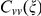 separated by a distance 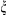, weighted by the competition kernel 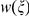 which describes how the interaction strength diminishes with the distance between pairs of competitors, and further weighted by the competition coefficient 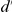. The final term deals with deaths through predation, and it has a similar form to the competition term, this time 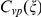 describing the density of predator-prey pairs separated by a distance 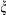, and the attack kernel 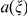 describing the intensity of predator foraging at a distance 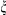 from a predator's nest. The dynamical system is actually still closely related to the non-spatial Lotka-Volterra model [21]; except now local correlations and the competition and attack kernels are included. Indeed, the classical Lotka-Volterra predator-prey equations can be recovered when spatial correlations vanish, i.e. 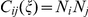, for all 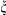; and this is expected to occur when movement/dispersal and competition/predation all occur over large spatial scales. The full system with the predator dynamics as well is presented in Appendix S1. The equation presented needs an additional competition term when a mutant is included (see below, eq. 2), and since the spatial patterns change after individual birth, death and movement events, the pair densities 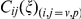 change over time. Additional equations are needed to describe their dynamics (Appendix S1). Movement events, and movement parameters such as dispersal rate and range, enter only in the pair densities equations, because they change only the expected spatial distribution of organisms across the landscape, and not the densities directly. From equation 1 or the IBM, one can derive the invasion fitness of a mutant prey in a resident population at equilibrium
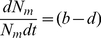
(2a)

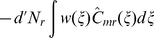
(2b)

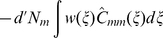
(2c)

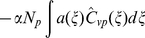
(2d)


All 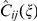 terms are normalized second moments, also called pair correlation functions, defined as 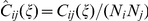. In general, we find that 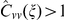 for small distances 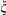 and 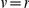 or 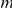, which means the prey is aggregated in space; and 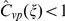 for small 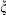, which means predator-prey pairs are more likely to be separated by some distance than what we would expect if they were distributed at random [21]. 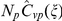 is the expected density of predators at a distance 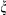 from a randomly chosen prey individual.

The second term (2b), incorporates deaths that are caused by local competition with the resident with the integration taking into account the expected density of resident competitors at all distances 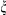, weighted by the competition kernel, 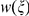. The third term (2c) similarly takes into account the neighborhood competition with other mutants, which even after the first births may be significant due to the local nature of dispersal [25].

### Invasion analyses

Using equation 2 and those of Appendix S1, we perform a series of invasion analyses to investigate the evolution of dispersal rate. Invasion analyses proceed by allowing a resident predator and prey community to reach its ecological equilibrium before introducing at low density a mutant prey having the same ecological parameters as the resident prey, except that it differs in its dispersal rate. Since we assume there is no explicit trade-off in dispersal rate with any other parameter in the model, selection for or against dispersal depends only on the relative importance of competition and predation in the mutant invasion fitness (see next section). The invasion analyses allow the production of pairwise-invasibility plots (PIPs) showing which phenotypic trait values can invade into a community dominated by one other dispersal trait value, over a broad range of resident and mutant trait values. From PIPs it is possible to visualise the expected evolutionary end-point, and how the trait subsitution sequence might proceed. Our approach is essentially a spatial extension to the adaptive dynamics approach [22], using moment equations. We verify some of the main results with simulations using the IBM (Figure S2).

## Results

Overall, we find that an increased predation intensity, as well as an increased heterogeneity in the spatial pattern of predation pressure selects for less dispersive prey.

### Intense predation selects against dispersal

**Figure 2:**
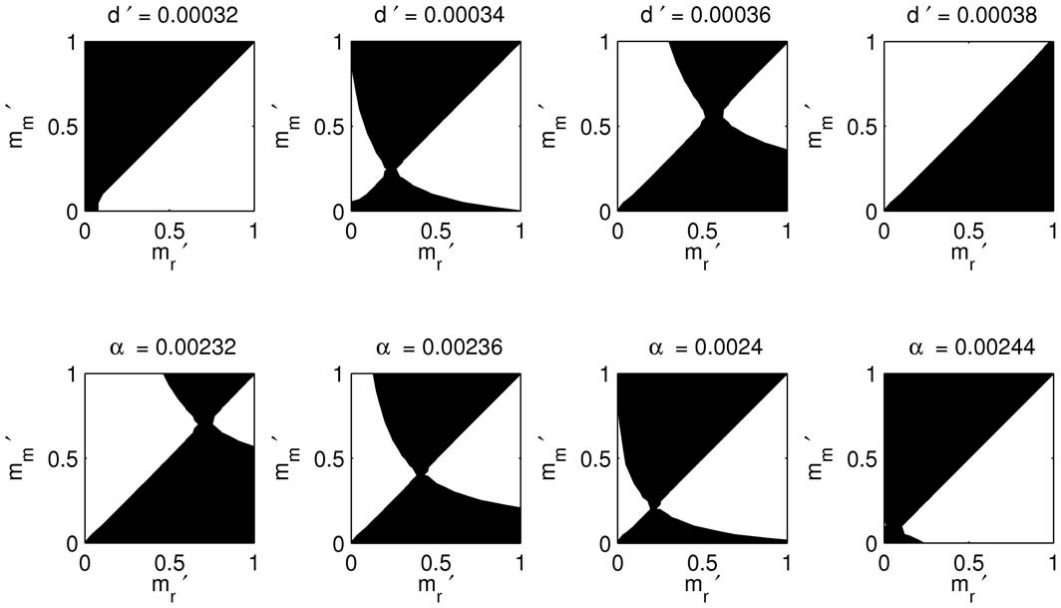
The effects of competition and predation intensity on the evolutionarily stable adult prey dispersal rate. Pairwise invasibility plots (PIPs) computed from the moment equations, for a gradient of competition rate 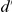 (upper row) and predation rate 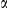 (lower row). White colouring indicates the mutant invades, and black that the mutant loses (does not invade). On the x-axis is represented the resident dispersal rate (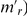 and on the y-axis the mutant dispersal rate (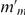). The ESS dispersal rate 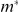 is located at the intersection between black and white parts of the plane, along the diagonal. It is also convergence stable, in that it can be attained in a series of small ‘mutational’ steps. The first row shows that 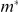 increases with prey competition strength, while the second row shows 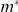 to decrease with predation rate. Parameters held constant are 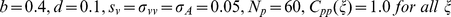. First row 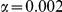, second row 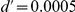. Here there are no post-natal predator movements (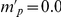).

**Figure 3:**
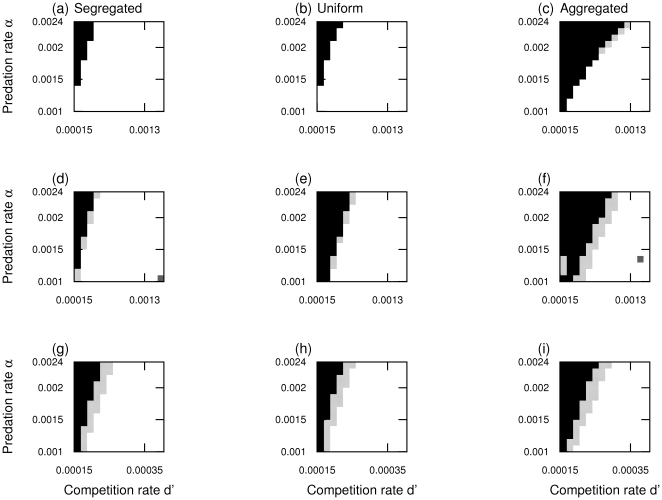
The effect of predator spatial pattern on the evolution of adult prey dispersal rate. These plots have been obtained by performing only two invasions for each parameter combination, corresponding to the opposite top left and bottom right corners of the PIP plots of Figure 2, for 3 predator spatial patterns (segregated, uniform, aggregated). The color represents the evolutionary outcome, white = selection for maximum dispersal, black = selection for no adult dispersal, gray = existence of an intermediate ES dispersal rate. The first row represents results obtained in one dimension, without predator demography. The second row presents the results with the predator demography. The third row depicts results obtained in two dimensions, without predator demography. Detailed parameters. General parameters are 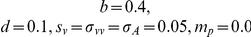 (a–b–c) No feedbacks, one dimension. 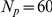. Uniform case, 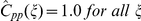, Segregated 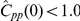, Aggregated 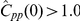. The values of the spatial autocorrelations are those that would have been obtained, if feedbacks were included (see below for the parameter values of predator demographic rates). (d–e–f) Demographic feedbacks, one dimension. Predator parameters 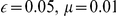. Specific parameters (d) 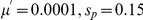 (e) 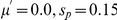 (f) 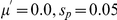 (g–h–i) Two dimensions (no demography) 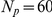.

As shown in Figure 2 (upper row), for a fixed predation rate, the evolutionarily stable (ES) dispersal rate increases when competition strength increases, which confirms earlier theoretical results [1]. In contrast, when fixing competition but increasing the predation rate (Figure 2 lower row), the ES dispersal rate decreases with the predation rate. We note that in the parameter regions studied, the boundary between very low and very high ES prey dispersal rates can be very narrow, meaning small changes to prey competition rates, or predation rates can greatly change the ES dispersal rate (Figure 2). In Figure 3, we cover broader regions of parameter space, so that only a thin strip of parameter space exhibit an ESS, separating runaway selection for or against dispersal (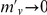 or 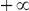). We also show that the results are not sensitive to the dimensionality of space (1 or 2), and most importantly, that when predators reproduce according to prey density (i.e. have a numerical response, and hence, variable numbers), the same selective pressures for dispersal are observed: more predation also selects, all other things being equal, for less dispersive prey individuals.

We explain these results using the difference between the mutant and resident fitnesses, which is expressed as
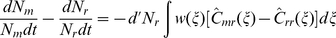
(3a)

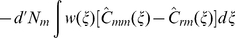
(3b)

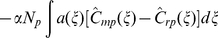
(3c)and the mutant has higher fitness when (eq. 3) is positive. The first (eq. 3a) and second (eq. 3b) competition terms are increased when 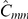 is lowered and 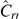 is increased; that is, when the mutant is less aggregated than the resident. Consequently, if 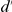 is high and competition terms dominate the fitness difference, the mutant wins by lowering its spatial autocorrelation and the resulting kin competition, and selection is for more dispersive individuals. However, when 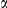 is large, and the predation term (eq. 3c) dominates, the fitness difference is increased when 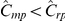, that is when spatial segregation between mutant prey and predator is higher than between resident prey and predator. This happens when prey dispersal is low; spatially heterogeneous predation selects against dispersal.

### Small predator home ranges and clustered predators select against prey dispersal

**Figure 4:**
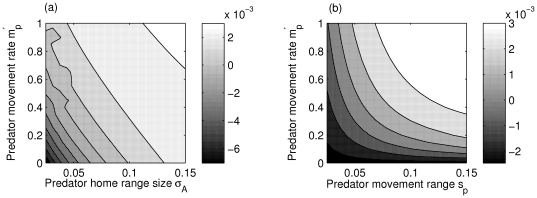
Effect of predator space-use parameters (movement rate, home range size) on adult prey dispersal rate. We present the invasion fitness of a dispersive mutant prey in a non-dispersive resident population (a convenient index of selection for dispersal that has been confirmed by more detailed PIPs) as a function of (a) predator home range size 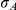 and predator movement rate 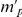; and (b)as a function of average predator dispersal distance (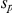) and predator movement rate 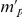. White indicates selection for dispersal (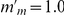, maximal value), and black against (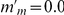), while gray values around zero fitness are an intermediate zone where there actually is a positive ES dispersal rate (as shown in Figure 2). Parameters held constant are 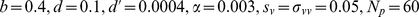. In (a) 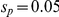 and in (b) 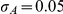. Invasion fitness was computed using the moment approximation.

When decreasing the home range size of predator individuals (starting from large overlapping home ranges), we progressively arrive at a spatially heterogeneous (autocorrelated) spatial pattern of predation risk (see Figures S3, S4 for illustrations). Figure 4a shows how decreasing the predator home range size (increased autocorrelation in predation) tends to select for less dispersal, while moving predators tend to select for increased dispersal. This is because when the home range or movement rate of predators is large, the point pattern of predators becomes less important in determining the losses of mutants through predation, and the mutant fitness is determined more by the competition terms of eqs. (3a) and (3b). In this case longer/more frequent dispersal is selected as it carries a low cost of extra predation since predators can already reach most prey individuals, yet helps avoiding much more kin competition because prey become less clustered in space. Increasing the spatial scale of predator movement is similar to increasing predator movement rate, although both need to be relatively large to select for significantly dispersive prey individuals (Figure 4b). Similarly, aggregated predators tend to select for lower dispersal rates in the prey (Figure 3) than segregated or uniformly distributed (i.e. complete spatial randomness) predators. This is because aggregated predators generate areas with low predation pressure (‘refuges’), and any prey individual born into these regions is better off staying where it is, unless competition is very high.

### Does selection lead to larger population sizes?

Here, we relate the selective pressures on prey adult dispersal rate to predator home range size and their effects on the prey population size (given a typical average prey competition and dispersal distance). In the case without feedback (i.e. without a predator numerical response to prey density) the model is analogous to models with spatially heterogeneous mortality [12], [13], [26]. When the feedback is present however, we have a more classical predator-prey model with two species interacting.

**Figure 5:**
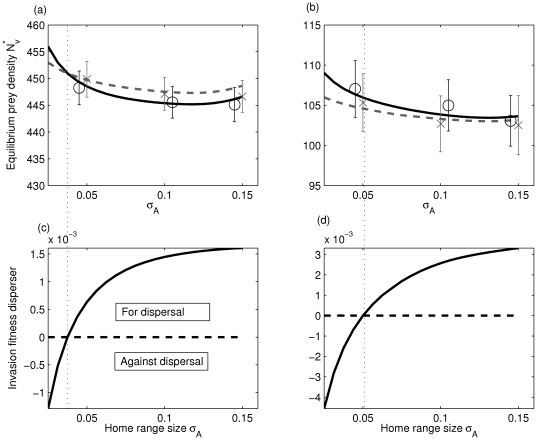
The effect of the evolutionarily stable prey dispersal strategies (adult dispersal rate) on the prey population sizes, for various predator home range sizes. In the upper panels (a) and (b) the equilibrium density of a resident (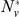) non-dispersive type (resp. dispersive) is represented with a filled line (resp. a dashed line), both when the predator numerical response is absent (a) and present (b). In the lower panels, we show an index of the selective pressure for dispersal, the invasion fitness of a dispersive mutant (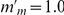) in a non-dispersive population (filled lines). The zero fitness value is shown with a horizontal dashed line, and separates selection against dispersal (below) versus selection for dispersal (above). The thin dotted line separates in all panel parameter regions selecting for and against dispersal. The case without a predator numerical response is presented in (c), while the numerical response is added in (d). Parameters: 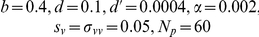. In the right column (b–d), additional predator parameters generating the demography take positive values; 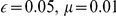. The solid lines are computed using the moment approximations, and the open circles and crosses in (a) and (b) are the landscape densities of prey averaged over 50 realisations of the IBM on a 1D line of length 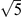.

We first consider the case without predator demography (Figure 5a), where it is shown that prey population size is maximised by an absence of dispersal only when predator home range size is very small. Prey population sizes are maximised by the selected adult dispersal strategy, as shown by the correspondance between Figures 5a and 5c; e.g. when dispersal is selected it leads to a larger population size.

When predator demography is present, that is, when predators have numerical responses to prey variation in local numbers, we obtain the same selective pressures as when predator density is constant (Figure 5d). Selection for prey dispersal occurs when prey competition is small-ranged or when the predator home range size is large (Figure 5d), or when predators are highly mobile (not shown). However, increasing prey dispersal leads to lower prey population sizes (Figure 5b) because it feeds predators that produce in turn more predators, therefore depressing the prey population in the long-run. Hence, when predator demography occurs on the same population dynamics timescale as the prey's, increased prey dispersal might be favoured by natural selection because of high kin competition (e.g. when the spatial scale of competition is small relative to the spatial scale of predation), although such a strategy will ultimately depress the overall prey population size due to the predator numerical response.

Even when predator numbers are constant, it is sometimes possible for the selected dispersal strategy to lead to slightly lower prey population sizes (Barraquand and Murrell, unpublished data). However, this only happens in the small region of parameter space that separates runaway selection for or against adult dispersal, where there is a positive and finite ES dispersal rate (as in Figure 2). In contrast, when the predator has a numerical response, selection for dispersal always lowers the prey population size.

## Discussion

We have shown that under the assumption of a negative fine-scale segregation between predators and prey, arising in our case because of prey depletion by localized predators (e.g. central-place foragers [23]), increasing the intensity of predation ultimately leads to selection against dispersal, even when kin competition is present. Moreover, increasing the spatial autocorrelation of the predation pressure, either by decreasing the predator home range size or increasing the autocorrelation of predators' nests, increases the selection against dispersal. The results apply to prey (adult) dispersal rate but also to prey natal dispersal distance (Figure S2), irrespective of whether predator demography is considered or not. However, a predator numerical response to prey density, while not changing the selective pressures, changes the outcome of the selected strategy for the prey population as a whole. In this case, increased prey dispersal can lead to lower prey population sizes and higher predator population sizes. More prey dispersal leads to a lower spatial segregation between predator and prey, and with a numerical response in the predator, in the long term this leads to a lowering of the landscape-level density of the prey. This is reminiscent of the tragedy of the commons [17], where individuals driven by self-interest end up destroying or degrading their public good. In our model the public good is enemy-free space, and dispersive prey individuals feed predators that produce in turn more predators, thereby increasing the proportion of space filled with predators. This detrimental effect of individual dispersal decreases when factors weakening the feedback are introduced, such as direct predator competition, in which case the situation is very close to when predators do not have a numerical response (Barraquand and Murrell, unpublished results).

Our model strengthens the argument for a negative effect of spatial variability on dispersal propensity [1], [8], [10] and extends it to interactive predator-prey systems. This is because it pays individuals to stay with related individuals (siblings and offspring in our model) in high quality patches (in our case areas of low predation risk), a process termed ‘habitat association’ by Bolker [26]. Increasing predator dispersal rate or range, which both decreases spatial variability and increases temporal variability from a prey point of view, tends to select for increased prey dispersal, and these results are in line with current theory on the evolution of dispersal in heterogeneous landscapes [13]. We note, however, that the shift in selection regimes observed in our model (selection either for or against adult prey dispersal) is largely a consequence of the absence of additional costs to dispersal; introducing trade-offs between parameters could produce a smoother transition. We did not include these additional costs because they might obscure the selective pressure created by predation in some regions of parameter space, but investigating how trade-offs between dispersal and other parameters might change our results is a topic worthy of further attention.

Another limitation of the model worth mentioning is the spatial scale of interaction and movements. In many of our analyses interaction ranges are considered the same for prey and predator, though of course, we relax the assumption when predator home range size is varied. While it seems feasible that the prey dispersal range could be equal to (or slightly below) the predator home range size, the prey competition range should be thought of representing exploitative competition - rather than direct interference - for its value to be biologically meaningful. Indeed, direct interference between two prey individuals is unlikely to occur at the same spatial scale as predator space use. It might be desirable in future work to vary more the spatial scales than we did; for instance with predator home range size several orders of magnitude above the typical prey dispersal and interaction distances, which will prove computationally challenging with the integrodifferential equations used here. That being said, our model still applies to many predator-prey systems once prey-prey competition is understood in a wide sense, and even other ecological contexts than predation. The effect of predation on dispersal in our model is indeed rather similar to a habitat disturbance (sensu [27]), which suggests that more spatially correlated disturbances can decrease the selection for dispersal, since more correlated predator movements select for lower prey dispersal rates.

Using a model tailored to explain the effect of nest predation on adult bird dispersal, [28] show that when predator home ranges are small or medium-ranged, there is no advantage to long-range adult prey dispersal. This is consistent with the results presented here, since the negative spatial correlation that arises from predators having a restricted home range selects against prey dispersal. Central-place foraging birds of prey are an archetypical example of localized predators generating this kind of selective pressure against prey dispersal [29]. This should equally be expected in territorial mammalian predators that have dens, such as in canids where a negative spatial correlation with their ungulate prey has previously been observed [30] and confirmed by modeling [31]. We therefore expect this selective pressure to be a robust pattern in animals preyed upon by birds and mammals using nests or dens, or exhibiting home range behaviour, and in many other prey species in which predators use refuges, which constrain space use in the same manner [32].

In contrast to our main results, Savill and Hogeweg [33] found, using spatial predator-prey models, that in the presence of predator-prey travelling waves caused by non-linear interactions, selection always leads to increased prey (and predator) dispersal. These waves emerge in their case from the handling time of predators that generates a saturation of attack rates with respect to prey local density. The presence of travelling waves means both predator and prey numbers locally oscillate, and because the predators lag only slightly behind the prey in space and time [34], [35], any negative correlation in space is likely to be weak which, as we show, tends to select for more or longer dispersal in the prey. In addition, spatially asynchronous temporal variation selects for dispersal, and the local oscillations are necessarily asynchronous to some extent for a wave to exist. In other words, when there are asynchronous temporal oscillations in abundance, if you are a prey individual in a sink that is becoming crowded with predators, you can disperse to a source nearby that is enemy-free.

We have not included non-linear predation rates at the local scale in our model nor another wave-generating mechanism, which means dispersal cannot be selected for by such processes. It would be interesting to perfom the analysis again with a local type II functional response to see whether this affects the results - it is actually technically demanding because of the explicit derivation of the equations from the IBM. On the one hand, it seems unlikely that intake rate saturation can change the sign of the spatial correlation between predator and prey, but on the other hand, it might generate a dilution of predation risk [36] which means prey might benefit from being spatially aggregated. In addition, the local type II response can generate oscillations; if prey dispersal range is larger than the spatial scale of local oscillations, dispersal might be selected for, because it allows prey to escape from temporarily risky habitats [33]. This suggests that predation might have different effects on prey movements at various spatial scales, which is consistent with empirical observations in a wolf-elk system [37]. Elk benefit from migrating because it lowers the predation pressure on a large (population) spatial scale, but at smaller scales, non-migrating elks in predator-rich habitat can benefit from moving less when they stay in human-dominated habitats that are avoided by wolves (i.e. there is a small-scale negative spatial correlation that favors less mobile elks).

More sophisticated predator behaviours, such as directed searching, might affect the evolutionarily stable strategy. For instance [38], found that in response to a randomly searching predator, prey should not move (this is of course true at a behavioural spatiotemporal scale, but the finding also applies at larger scale). In constrast, in response to a directed searcher exploiting prey aggregations, prey should blur their spatial pattern by moving on a large spatial scale [38]. Preliminary simulations including predator displacement of the home range center dependent on prey local density suggest that ‘informed’ predator movements can indeed select for more dispersal in the prey, which is in accordance with the fact that directed predator movement lowers the predator-prey segregation. This is reminiscent of Tinbergen et al. [39] who observed that, in response to predators using area-restricted search, prey individuals should avoid conspecifics and space out. However, another line of work suggests that prey should directly avoid predators [40]. Whether or not it is better to avoid conspecifics or predators when predators are efficient searchers is an open question, and is worthy of more attention both theoretically and empirically. In a recent paper, Poethke et al. [41] develop an empirically-motivated model (for aphids), that includes detection of recent predation by the prey. Their analyses show how dispersal rates that are dependent upon the predator density should be low if predator revisit rates are low. Investing in predator avoidance in their model is valuable only when substantial temporal correlation in predator movement exists; otherwise it is best not to move.

The suggested extensions to the model proposed above concentrate on incorporating feeding constraints, adaptive foraging, and avoidance behaviour; but there are other evolutionary forces at work, and these pertain to differences between the sexes and also to kin structure. The model presented here does not consider differences in dispersal pressure between sexes, and this is due in no small part due to the implicit assumption that the individuals are females, and that males are not limiting reproduction. Relaxing this assumption might yield more complex results. For instance, males might accept a higher predation risk if dispersing entails increased fitness benefits such as access to more females. This would clearly have important implications for gene flow within the population, but the genetic structure would need to be made explicit in order to see how the gene flow is affected by such sex biased dispersal.

In conclusion, we have shown that in cases where there is not much information about the predator distribution and predation pressure is spatially variable, it might pay a prey individual to stay put rather than risk dispersing into the ‘lion's den’. Our results have also shown once more how ecological and evolutionary dynamics may be intricately linked; whilst there appears to be no evolutionary suicide as has been found in a metapopulation model [42], our results do suggest that both predation itself, and the evolutionary response to predation may actually act to reduce the population density of the prey. Incorporation of other processes and behaviours, notably a plastic response that is dependent upon the current level of predation experienced, or directed prey movement away from high densities of predators, may alter some of the basic results shown here, by reducing the cost involved, and by allowing dispersing individuals to better avoid hungry predators. Nevertheless, we still expect that any spatially variable predation pattern with a negative spatial correlation between predators and prey, as generated by central-place foragers, will push selection against prey dispersal, since most prey individuals are born in relatively safe habitats.

## Supporting Information

Figure S1
**Confirmation of moment equation results using the IBM** Prey dispersal increases with strength of competition 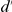 and decreases with of predation 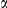. The colors depict the percentage of successful invasions by very dispersive mutants into weakly dispersive populations at a demographic equilibrium (stochastic fluctuations in numbers notwithstanding). Black color means dispersive mutants never invade while white implies they always do so. (a) presents the results for the evolution of adult dispersal rate (residents have 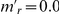 and mutants 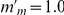, 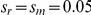), while (b) presents the results for the evolution of natal dispersal range (residents have 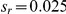 and mutant 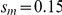, 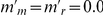). The parameters values are 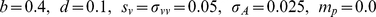, 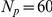, 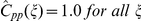. There is no predator demography here, so that 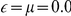. Note that here, it makes sense not to compute PIP plots because on the ranges of parameter values (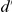 and 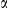) considered, there is no ESS dispersal parameter value (runaway selection either for or against dispersal). We see that the results obtained with the help of moment equations are also verified by the individual-based models.(TIFF)

Figure S2
**Dispersal range evolution** Pairwise invasibility plots for the evolution of dispersal range on a gradient of prey competition intensity 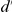. As prey competition increases, selection for dispersal increases (opposite results can be found when increasing predation intensity, and are similar to those of dispersal rate evolution). The difference here is that we actually observe a repellor point that goes down the diagonal of the PIP, instead of an ESS going up, as in Figure 2a. The colors are reversed when compared to Figure 2a. In other words, we observe the same overall selective pressures than for dispersal rate (runaway selection against dispersal under weak competition/strong predation) but things differ around the repellor point. When there is a repellor point, it means that around these parameter values, the outcome of the evolutionary game is determined by the initial value of the dispersal range. If it is small and below the repellor, then selection will lead to an even smaller dispersal range, if it is large, selection will lead to an even larger dispersal range. Some regions of parameter space exhibit both an ESS and a repellor, but they tend to be quite narrow and the IBM often predicts extinction for these values. The parameters values used in this figure are 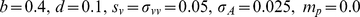, 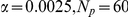, 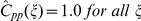.(TIFF)

Figure S3
**Snapshot of the IBM in 2 dimensions.** The upper right panel has labels for X and Y spatial coordinates, and the three other plots are similarly constructed. All other labels indicate parameters that change between panels. The upper row represents small predator home ranges (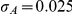), lower row larger predator home ranges (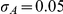); in the left column adult prey dispersal rate is null (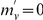), right column adult prey dispersal rate is large (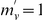). Predators are depicted as circles, prey items as stars, and yellow (resp. red) shading represents low (resp. high) predation risk for prey. Parameters used: 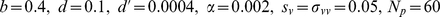. Constant predator numbers, no predator movement nor birth/death.(TIFF)

Figure S4
**Space-time plots for the IBM in one dimension.** The upper right panel has labels for spatial dimension (X-axis) and temporal dimension (Y-axis), and the three other plots are similarly constructed. All other labels indicate parameters that change between panels. The upper row represents small predator home ranges (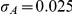), lower row larger predator home ranges (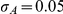); in the left column adult prey dispersal rate is null (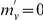), right column adult prey dispersal rate is large (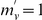). Predators are depicted as unfilled circles, prey items as black filled circles. Parameters used: 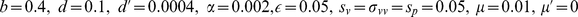.(TIFF)

Appendix S1 **Supplementary methods** Appendix S1 describes the methods used to introduce mutants in the predator-prey model, simulate the IBM, and numerically integrate the moment equations.
(PDF)

## References

[1] Johnson M, Gaines M (1990). Evolution of dispersal: theoretical models and empirical tests using birds and mammals.. Annu Rev Ecol Evol Syst.

[2] Bowler D, Benton T (2005). Causes and consequences of animal dispersal strategies: relating individual behaviour to spatial dynamics.. Biol Rev.

[3] Ronce O (2007). How does it feel to be like a rolling stone? Ten questions about dispersal evolution.. Annu Rev Ecol Evol Syst.

[4] Hamilton W, May R (1977). Dispersal in stable habitats.. Nature.

[5] Holt R, McPeek M (1996). Chaotic population dynamics favors the evolution of dispersal.. Am Nat.

[6] Cadet C, Ferriére R, Metz J, van Baalen M (2003). The evolution of dispersal under demographic stochasticity.. Am Nat.

[7] Murrell D, Travis J, Dytham C (2002). The evolution of dispersal distance in spatially-structured populations.. Oikos.

[8] Hastings A (1983). Can spatial variation alone lead to selection for dispersal?. Theor Popul Biol.

[9] McPeek M, Holt R (1992). The evolution of dispersal in spatially and temporally varying environments.. Am Nat.

[10] Travis J, Dytham C (1999). Habitat persistence, habitat availability and the evolution of dispersal.. Proc Roy Soc (Lond) B.

[11] Travis J (2001). The color of noise and the evolution of dispersal.. Ecol Res.

[12] Bolker B, Cantrell S, Ruan S, Cosner C (2009). Evolution of dispersal scale and shape in heterogeneous environments: A correlation equation approach.. Spatial Ecology.

[13] North A, Cornell S, Ovaskainen O (2011). Evolutionary responses of dispersal distance to landscape structure and habitat loss.. Evolution.

[14] Weisser W (2001). The effects of predation on dispersal..

[15] Roth T, Lima S (2007). Use of prey hotspots by an avian predator: purposeful unpredictability?. Am Nat.

[16] Clobert J, Danchin E, Dhondt A, Nichols J (2001). Dispersal..

[17] Rankin D, Bargum K, Kokko H (2007). The tragedy of the commons in evolutionary biology.. Trends Ecol Evol.

[18] Bolker B, Pacala S (1997). Using moment equations to understand stochastically driven spatial pattern formation in ecological systems.. Theor Popul Biol.

[19] Dieckmann U, Law R, Dieckmann U, Law R, Metz J (2000). Relaxation projections and the method of moments.. The geometry of ecological interactions: symplifying spatial complexity.

[20] Geritz S, Kisdi E, Meszéna G, Metz J (1998). Evolutionarily singular strategies and the adaptive growth and branching of the evolutionary tree.. Evol Ecol.

[21] Murrell D (2005). Local spatial structure and predator-prey dynamics: Counterintuitive effects of prey enrichment.. Am Nat.

[22] Le Gaillard J, Ferriere R, Dieckmann U (2003). The adaptive dynamics of altruism in spatially heterogenous populations.. Evolution.

[23] Moorcroft P, Lewis M (2006). Mechanistic home range analysis..

[24] McCauley E, Wilson W, de Roos A (1996). Dynamics of age-structured predator-prey populations in space: asymmetrical effects of mobility in juvenile and adult predators.. Oikos.

[25] Murrell DJ (2010). When does local spatial structure hinder competitive coexistence and reverse competitive hierarchies?. Ecology.

[26] Bolker B (2003). Combining endogenous and exogenous spatial variability in analytical population models.. Theor Popul Biol.

[27] Hiebeler D, Morin B (2007). The effect of static and dynamic spatially structured disturbances on a locally dispersing population.. J Theor Biol.

[28] Powell L, Frasch L (2000). Can nest predation and predator type explain variation in dispersal of adult birds during the breeding season?. Behav Ecol.

[29] Shultz S, Noë R (2002). The consequences of crowned eagle central-place foraging on predation risk in monkeys.. Proc Roy Soc (Lond) B.

[30] Mech L (1977). Wolf-pack buffer zones as prey reservoirs.. Science.

[31] White K, Murray J, Lewis M (1996). Wolf-deer interactions: a mathematical model.. Proc Roy Soc (Lond) B.

[32] Chase J (1998). Central-place forager effects on food web dynamics and spatial pattern in northern california meadows.. Ecology.

[33] Savill N, Hogeweg P (1999). Competition and dispersal in predator-prey waves.. Theor Popul Biol.

[34] Sherratt J, Smith M (2008). Periodic travelling waves in cyclic populations: field studies and reaction-diffusion models.. J R Soc Interface.

[35] Hagen S, Jepsen J, Schott T, Ims R (2010). Spatially mismatched trophic dynamics: cyclically outbreaking geometrids and their larval parasitoids.. Biol Lett.

[36] Turchin P, Kareiva P (1989). Aggregation in Aphis varians: an effective strategy for reducing predation risk.. Ecology.

[37] Hebblewhite M, Merrill E (2007). Multiscale wolf predation risk for elk: does migration reduce risk?. Oecologia.

[38] Mitchell W, Lima S (2002). Predator-prey shell games: large-scale movement and its implications for decision-making by prey.. Oikos.

[39] Tinbergen N, Impekoven M, Franck D (1967). An experiment on spacing-out as a defence against predation.. Behaviour.

[40] Hauzy C, Hulot F, Gins A, Loreau M (2007). Intra-and interspecific density-dependent dispersal in an aquatic prey-predator system.. J Anim Ecol.

[41] Poethke H, Weisser W, Hovestadt T (2010). Predator-induced dispersal and the evolution of conditional dispersal in correlated environments.. Am Nat.

[42] Gyllenberg M, Parvinen K, Dieckmann U (2002). Evolutionary suicide and evolution of dispersal in structured metapopulations.. J Math Biol.

