# Supplementary figures and images for: Intense or Spatially Heterogeneous Predation Can Select against Prey Dispersal
Source: PLoS One. 2012 Jan 11;7(1):e28924. doi: 10.1371/journal.pone.0028924 (PMC3256147; doi:10.1371/journal.pone.0028924)

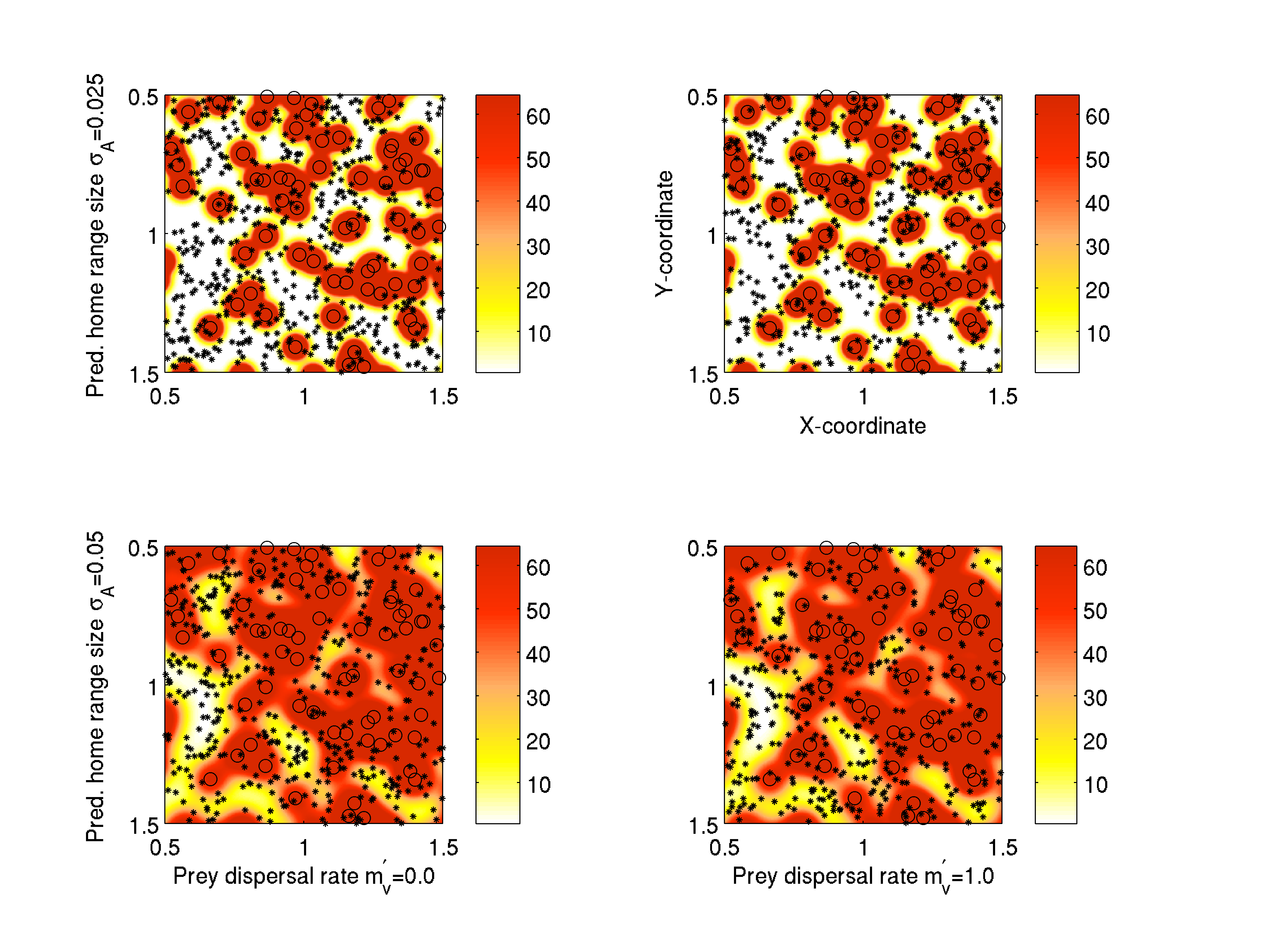

Supplement: Figure S3 — Snapshot of the IBM in 2 dimensions. The upper right panel has labels for X and Y spatial coordinates, and the three other plots are similarly constructed. All other labels indicate parameters that change between panels. The upper row represents small predator home ranges (), lower row larger predator home ranges (); in the left column adult prey dispersal rate is null (), right column adult prey dispersal rate is large (). Predators are depicted as circles, prey items as stars, and yellow (resp. red) shading represents low (resp. high) predation risk for prey. Parameters used: . Constant predator numbers, no predator movement nor birth/death. (TIFF) [file pone.0028924.s003.tif]
