# Supplementary material for: Intense or Spatially Heterogeneous Predation Can Select against Prey Dispersal
Source: PLoS One. 2012 Jan 11;7(1):e28924. doi: 10.1371/journal.pone.0028924 (PMC3256147; doi:10.1371/journal.pone.0028924)
Supplement: Appendix S1 — Supplementary methods Appendix S1 describes the methods used to introduce mutants in the predator-prey model, simulate the IBM, and numerically integrate the moment equations. (PDF) [file pone.0028924.s005.pdf]

F. Barraquand and D. Murrell. Appendix S1: Supplementary methods

1 The two-prey / one predator point process model and its covariances dynamics

In this appendix we describe the equations used to obtain the results presented in the paper. The basic form of the equations is very similar to those described in Murrell [1] where the one predator and one prey species model is described in greater detail. The difference is that here we introduce a mutant prey species (or phenotype) along with the resident prey species, and therefore we need a two prey and one predator model. We obtain roughly the same equations as in [1], but with the prey index v taking the values r, m, as well as a few other additional modifications that are described below.

The derivation of the equations from the IBM is rather cumbersome and lengthy, and is therefore not described here. We refer the reader to [2] for more details on these derivations using master equations in a competition context, and a mathematical derivation for this predator-prey model using a master equation approach can be obtained from the corresponding author upon request.

Individual-based model

Rates in the individual based model

The individual event rates do not change much with respect to [1]. For instance, using the resident death rate as an example, the main change is that it now also depends on competition with the mutant. Therefore, the death rate of a resident prey individual at location x is given by

$$D_r(x, x', p) = d + \underbrace{d' \int w_r(x'-x)[p_r(x') - \delta_x(x')]dx'}_{compet.\ with\ residents} + \underbrace{d' \int w_m(x'-x)p_m(x')dx'}_{compet.\ with\ mutants}$$
$$+ \underbrace{\alpha \int a(x'-x)p_p(x')dx'}_{predation}$$

where $\delta_x(x')$ refers to the Dirac delta function, which is used to remove self-competition at $x' = x$, and $p_i(x'),\ i = r, m, p$ are sums of Dirac delta functions representing the point patterns of resident, mutants, and predators, respectively. The first term is simply the constant per capita rate of death. The second term sums over all areas around an individual, weighting the competitive effect of other resident prey at all locations x' by the distance from the focal individual; this being described by the competition kernel $w(x'-x)$. The Dirac delta function removes the effect of the focal individual on itself as we assume there is no self-competition. The third term repeats this process, but now for all mutant individuals in the neighborhood of the target resident prey. The final term then considers the probability per unit time that the individual will be eaten by a predator within its neighborhood; with the attack kernel $a(x'-x)$ describing how the attack rate is modified by the distance $x'-x$ between the target resident prey at location x and the density of predators at x'. The death rate for the mutant prey has the same form.

The predator birth rate needs some modifications as well as it now includes the two prey species

$$B_p(x, x', p) = \epsilon\alpha m_p(x'-x) \int a(x''-x)[p_r(x'') + p_m(x'')]dx''$$

Essentially the predator birth rate is the same as in [1]; with the terms after the integral taking into consideration the rate at which resident and mutant prey at location x'' are attacked and eaten

by the predator, and this is described by the attack kernel $a(x'' - x)$. The terms before the integral are the product of a spatially averaged attack rate α, and conversion efficiency ϵ which determines the probability that a prey item eaten will be converted into a newborn predator. The predator dispersal kernel, $m_p(x' - x)$, then describes the probability that a newborn predator lands at a location x' around its parent.

Simulation of the individual-based model

The algorithm used to simulate the IBM (to confirm the results of the moment approximation as well as produce Fig. 4) is based on the following principle (discrete event simulator for a generalised continuous-time Markov chain)

1. Compute the sum S of all event rates for all individuals of all species.
2. Compute the time to next event with an exponential distribution, whose mean is $1/S$. This means that when there are many individuals interacting quite strongly, S is large and the waiting time between events is short; but when there are few individuals and/or interactions and birth/death rates are slow, then the waiting times between events are longer.
3. Decide which event will happen. Let k be the type of event (b: birth, d: death, m: movement), i the index of the individual and s its species. E_{kis} is then the corresponding event rate, whose probability of occurring is E_{kis}/S. For instance, the 37th resident prey dies with probability $E_{d,37,r}/S$ where $E_{2,37,r} = d + d' \sum_{i=1, i \neq 37}^{\#preys} w(x_i - x_{37}) + \alpha \sum_{j=1}^{\#predators} a(x_j - x_{37})$. A simple comparison of cumulated probabilities to a uniform random number (between 0 and 1) then determines which event occurs (and to which individual).
4. Realise the event (i.e. move, or kill an individual, or create a new one in the case of a birth)
5. Update all the rates according to the new configuration (point pattern), and return to step 1.

Similar algorithms have been employed by [1, 3, 4], and allow for a unequivocal simulation of the dynamic point process.

First moments

The model for the dynamics of the landscape densities is (including predator demography)

$$\frac{dN_r}{dt} = (b - d)N_r - d' \int C_{rr}(\xi) w_r(\xi) d\xi - d' \int C_{rm}(\xi) w_m(\xi) d\xi - \alpha \int C_{rp}(\xi) a(\xi) d\xi \quad (1)$$

$$\frac{dN_m}{dt} = (b - d)N_m - d' \int C_{mm}(\xi) w_m(\xi) d\xi - d' \int C_{mr}(\xi) w_r(\xi) d\xi - \alpha \int C_{mp}(\xi) a(\xi) d\xi \quad (2)$$

$$\frac{dN_p}{dt} = \epsilon\alpha \int a(\xi)[C_{rp}(\xi) + C_{mp}(\xi)] d\xi - \mu N_p - \mu' \int C_{pp}(\xi) w_p(\xi) d\xi \quad (3)$$

In both equations (4) and (5), the first terms takes into consideration the net effect of the density independent births and deaths on the dynamics of resident and mutant prey populations, respectively. The second terms consider the losses due to neighborhood conspecific competition, and the third terms losses due to neighborhood competition between the two phenotypes. The final terms in (4) and (5) take into account deaths caused by neighborhood predation. In equation (6), the first term considers the increase in predator density due to neighborhood dependent predation of both resident, and mutant prey. The second term takes into account the losses of predators through the density independent death rate, and the final term takes into consideration losses of predators through neighborhood competition.

Second moments

Predator-prey cross-covariance

We illustrate this from the resident prey point of view (the mutant equation can be obtained by replacing r indexes by m and vice versa). The equation doesn't really change from [1] who considers the usual one predator and one prey species model, except that density-dependent death (prey-prey competition) can now occur also because of the mutant species, and also that predator birth (adding a new resident-predator pair) can occur because of mutant consumption.

$$\frac{dC_{rp}}{dt} = \underbrace{\int bm_v(\xi')C_{rp}(\xi+\xi')d\xi'}_{prey\ birth} \underbrace{-dC_{rp}(\xi)}_{prey\ di\ death} \tag{4}$$

$$\underbrace{-d'\int T_{rpr}(\xi,\xi')w_r(\xi')d\xi' - d'\int T_{rpm}(\xi,\xi')w_m(\xi')d\xi'}_{prey\ dd\ death} \tag{5}$$

$$\underbrace{-\alpha\int a(\xi')T_{rpp}(\xi,\xi')d\xi' - \alpha a(\xi)C_{rp}(\xi)}_{predation} \tag{6}$$

$$\underbrace{+m'_r\int m_v(\xi')C_{rp}(\xi+\xi')d\xi' - m'_rC_{rp}(\xi)}_{prey\ move} \tag{7}$$

$$\underbrace{+\epsilon\alpha\int\int [T_{prr}(-\xi+\xi',\xi'') + T_{prm}(-\xi+\xi',\xi'')]m_p(\xi')a(\xi'')d\xi''d\xi'}_{pred\ birth} \tag{8}$$

$$\underbrace{+\epsilon\alpha\int C_{pr}(-\xi+\xi')m_p(\xi')a(-\xi+\xi')d\xi'dx}_{pred\ birth} \tag{9}$$

$$\underbrace{-\mu C_{pr}(-\xi) - \mu'\int T_{prp}(-\xi,\xi')w_p(\xi')d\xi'}_{pred\ death} \tag{10}$$

$$\underbrace{m'_p\int m_p(\xi')C_{rp}(\xi'+\xi)d\xi' - m'_pC_{rp}(\xi)}_{pred\ move} \tag{11}$$

We illustrate here how all these terms are related to the previous events (dd: density-dependent; di: density-independent); a geometric interpretation of all terms can be derived, see the Appendix in [1] for more details.

Prey autocovariance

We show the prey autocovariance dynamics for the resident (just replace r by m and reciprocally to obtain the mutant equations)

$$\frac{dC_{rr}(\xi)}{dt} = bm_r(\xi)N + b\int C_{rr}(\xi+\xi')m_r(\xi')d\xi' \tag{12}$$

$$+m_r\int C_{rr}(\xi+\xi')m_r(\xi')d\xi' - m_r C_{rr}(\xi) \tag{13}$$

$$-dC_{rr}(\xi) - d'w_r(\xi)C_{rr}(\xi) \tag{14}$$

$$-d'\int w_m(\xi')T_{rrr}(\xi,\xi')d\xi' - d'\int w_r(\xi)T_{rrm}(\xi,\xi')d\xi' \tag{15}$$

$$+\alpha\int a(\xi')T_{rrp}(\xi,\xi')d\xi' \tag{16}$$

$$+ < \xi \to -\xi > \tag{17}$$

$< \xi \to -\xi >$ signify that all terms have to be doubled, replacing ξ by $-\xi$. Although the integrodifferential equations seems complicated, all terms have a geometric interpretation, see the Appendix in [1] for more details.

Prey resident-mutant cross-covariance

The prey resident-mutant covariance is a new element wrt the 1 prey / 1 predator model described in [1]

$$\frac{dC_{rm}(\xi)}{dt} = +b\int m_r(\xi)C_{rm}(\xi+\xi')d\xi' \tag{18}$$

$$-dC_{rm}(\xi) - \Big\{d'w_m(\xi)C_{rm}(\xi)d\xi \tag{19}$$

$$+d'\int w_r(\xi')T_{rmr}(\xi,\xi')d\xi' + d'\int w_m(\xi')T_{rmm}(\xi,\xi')d\xi'\Big\} \tag{20}$$

$$-\alpha\int a(\xi')T_{rmp}(\xi,\xi')d\xi' \tag{21}$$

$$+m_r'\Big\{\int m_v(\xi')C_{rm}(\xi+\xi')d\xi' - C_{rm}(\xi)\Big\} \tag{22}$$

$$+ < \xi \to -\xi, r \leftrightarrow m > \tag{23}$$

Note that in the correction term for density-dependent deaths, the kernel applies to the mutant.

Predator autocovariance

$$\frac{dC_{pp}(\xi)}{dt} = \epsilon\alpha \int\int T_{ppr}(\xi+\xi',\xi'')m_p(\xi')a(\xi'')d\xi' d\xi'' \quad (24)$$

$$+\epsilon\alpha \int m_p(-\xi)a(\xi'')C_{pr}(\xi'')d\xi'' \quad (25)$$

$$+\epsilon\alpha \int\int T_{ppm}(\xi+\xi',\xi'')m_p(\xi')a(\xi'')d\xi' d\xi'' \quad (26)$$

$$+\epsilon\alpha \int m_p(-\xi)a(\xi'')C_{pm}(\xi'')d\xi'' \quad (27)$$

$$m_p'\Big\{\int m_p(\xi')C_{pp}(\xi'+\xi)d\xi' - C_{pp}(\xi)\Big\} \quad (28)$$

$$-\mu C_{pp}(\xi,p) \quad (29)$$

$$-\mu' \int T_{ppp}(\xi,\xi')w_p(\xi')d\xi' \quad (30)$$

$$-\mu' w_p(\xi)C_{pp}(\xi) \quad (31)$$

$$+ < \xi \to -\xi > \quad (32)$$

Here predator movement is density-independent, and is realised according to the movement kernel $m_p(\xi)$ whose average distance moved is s_p.

Numerical details of the implementation

The numerical integration was done with an adaptive Euler scheme using steps of size $dt = 0.1$ (resp. 0.2 and 0.5) when the maximum of the first derivatives was > 0.1 (resp. > 0.01 and < 0.01). The equilibrium was considered reached when all the first derivatives were < 0.001. We considered the invasion was successful if the mutant fitness was superior to that of the resident at a precision of 10^{-5} after 20 time units (sufficient given the high prey growth rates involved). The spatial window used for the spatial integration was $[-0.5; +0.5]$ and is described by a grid with each grid cell having a width of 0.02 units, giving rise to 51 grid cells in the one dimensional case (25 cells in either direction, and one cell for the the pair density at distance 0).

In the dynamics for all of the spatial covariances, third moments arise whenever there is a density dependent process. These third moments are denoted by T, and describe the density of triplets. This means the dynamical system is not yet closed, and in order to do so, we approximate the third moments with a function of the first and second moments. This method is called moment closure, and is necessary since the dynamics of the third moments, if derived, would be functions of fourth moments; and the dynamics of the fourth moments functions of fifth moments; and so on. The moment closure we use to generate the results given in the main text is as follows:

$$T_{ijk}(\xi,\xi') = \frac{1}{5}\Big(4\frac{C_{ij}(\xi)C_{ik}(\xi')}{N_i} + \frac{C_{ij}(\xi)C_{jk}(\xi'-\xi)}{N_j} + \frac{C_{ik}(\xi')C_{jk}(\xi'-\xi)}{N_k} - N_iN_jN_k\Big) \quad (33)$$

References

[1] Murrell D (2005) Local spatial structure and predator-prey dynamics: Counterintuitive effects of prey enrichment. Am Nat 166: 354–367.

[2] Dieckmann U, Law R (2000) Relaxation projections and the method of moments. In: Dieckmann U, Law R, Metz J, editors, The geometry of ecological interactions: symplifying spatial complexity, Cambridge University Press, New York. pp. 412–455.

[3] Bolker B, Pacala S (1997) Using moment equations to understand stochastically driven spatial pattern formation in ecological systems. Theor Popul Biol 52: 179–197.

[4] Law R, Murrell D, Dieckmann U (2003) Population growth in space and time: Spatial logistic equations. Ecology 84: 252–262.
